# Inhibitory Effect of Bone Morphogenetic Protein 4 in Retinal Pigment Epithelial-Mesenchymal Transition

**DOI:** 10.1038/srep32182

**Published:** 2016-09-02

**Authors:** Haipei Yao, Hui Li, Shuai Yang, Min Li, Chun Zhao, Jingfa Zhang, Guotong Xu, Fang Wang

**Affiliations:** 1 Department of ophthalmology, Shanghai Tenth People’s Hospital, Tongji University, School of Medicine, Shanghai, China; 2 Tongji Eye Institute, Tongji University School of Medicine, Shanghai, China

## Abstract

Proliferative vitreoretinopathy (PVR), a serious vision-threatening complication of retinal detachment (RD), is characterized by the formation of contractile fibrotic membranes, in which epithelial-mesenchymal transition (EMT) of the retinal pigment epithelium (RPE) is a major event. Recent studies suggest an important role of bone morphogenetic protein 4 (BMP4) in the suppression of fibrosis. In this study, we aimed to investigate the role of BMP4 in the pathological process of PVR, particularly in the EMT of RPE cells. We found that BMP4 and its receptors were co-labelled with cytokeratin and α-SMA positive cells within the PVR membrane. Moreover, the mRNA and protein expression levels of BMP4 were decreased whereas BMP4 receptors ALK2, ALK3 and ALK6 were increased during TGF-β-induced EMT in primary RPE cells. Exogenous BMP4 inhibited TGF-β-induced epithelial marker down-regulation, as well as mesenchymal marker up-regulation at both the mRNA and protein levels in RPE cells. In addition, BMP4 treatment attenuated the TGF-β-induced gel contraction, cell migration and Smad2/3 phosphorylation. However, knockdown of endogenous BMP4 stimulated changes in EMT markers. Our results confirm the hypothesis that BMP4 might inhibit TGF-β-mediated EMT in RPE cells via the Smad2/3 pathway and suppress contraction. This might represent a potential treatment for PVR.

Proliferative vitreoretinopathy (PVR), the most common cause of surgical failure of rhegmatogenous retinal detachment (RD), is characterized by the formation of cellular membranes both on the retinal surface and within the vitreous cavity. The contraction of the membranes results in tractional retinal detachment^1^,^2^. Retinal pigment epithelial (RPE) cells on the epiretinal membranes (ERMs) are considered as key factors in PVR, as they undergo the epithelial-mesenchymal transition (EMT) process when triggered by vitreal cytokines^3^,^4^, such as transforming growth factor beta (TGF-β), bone morphogenetic proteins (BMPs), epidermal growth factor (EGF), fibroblast growth factor (FGF), hepatocyte growth factor (HGF) and WNTs^5^,^6^. TGF-β-induced EMT triggers epithelial cells to lose their epithelial phenotype and acquire mesenchymal properties. Loss of cell polarity and gain of migration is a change of cellular function during the EMT process^7^. Our previous study showed that TGF-β1 plays an essential role in the EMT process in human adult RPE cell lines (ARPE-19)^8^.

Bone morphogenetic proteins, the largest subfamily of the TGF-β superfamily, play a crucial role in specific physical and pathophysiological processes such as embryogenesis, skeletal formation and fibrosis^9^,^10^,^11^. Although first extracted from bone tissue, research has suggested changing their nomenclature from ‘Bone’ to ‘Body’ morphogenetic proteins because of their array of functions^11^. Bone morphogenetic proteins have been shown to play an essential role in eye development^12^,^13^. Moreover, the anti-fibrotic effects of BMPs make them attractive targets for the treatment of several diseases^14^. To date, more than 20 subtypes of BMPs have been identified in humans^15^. In terms of fibrosis, BMP2, BMP4 and BMP7 have garnered considerable attention^15^. BMP2 has been suggested to suppress TGF-β1-induced EMT in an *in vivo* model of renal fibrosis by attenuating Snail expression^16^. BMP7 reduces the endothelial-mesenchymal transition in a mouse model of heart failure^17^,^18^. Recombinant BMP7 reduces the severity of both acute renal injury and chronic model of diabetic nephropathy^19^,^20^. Interestingly, the role of BMP4 in fibrosis remains controversial. In the airway epithelium, BMP4 induces EMT and enhances cell migration^21^. In contrast, BMP4 is capable of blocking TGF-β2-stimulated fibronectin expression and extracellular matrix (ECM) production^22^.

In terms of signaling, TGF-β and BMPs act through two similar classes of receptors known as type I (TβRI, BMPR-I) and type II (TβRII, BMPR- II) receptors. Bone morphogenetic protein ligands bind the BMPR-II, which constitutively phosphorylates BMPR-I^23^. The following three type I receptors are preferentially bound by BMPs: activin receptor-like kinase (ALK)-2 (activin receptor type IA), ALK-3 (BMPR-IA), and ALK-6 (BMPR-IB)^24^. On the other hand, TGF-β binds to TβRII and activates TβRI to trigger downstream signalling^14^,^24^,^25^. TGF-β and BMPs counter-regulate each other and share similar downstream canonical sma and mothers against decapentaplegic (Smad) signalling pathways or non-canonical signalling pathways^26^. Thus, they keep the balance of normal biological activities. However, during EMT process, this balance is disrupted by the upregulation of TGF-β.

In this study, we investigated whether BMP4 plays a potential role in inhibiting the TGF-β-induced EMT in RPE cells. In addition, we look forward to developing a new drug for the treatment of PVR.

## Results

### BMP4 and cognate receptors expression within PVR membranes

**Figure 1:**
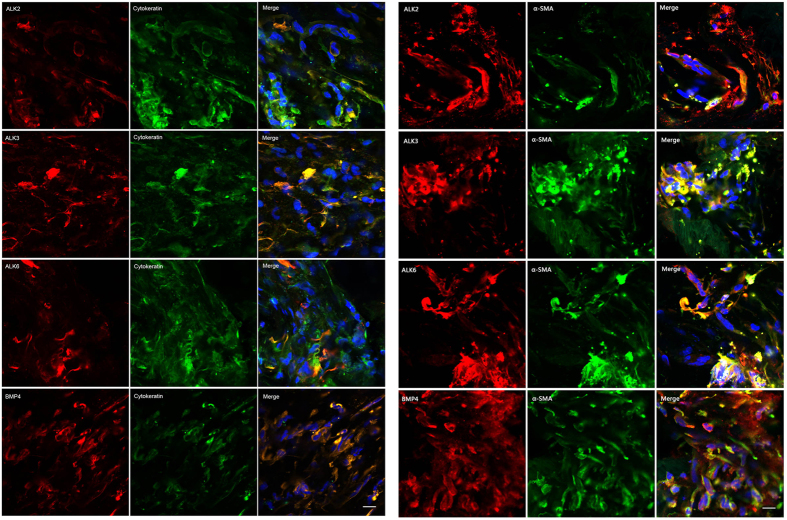
Expression of BMP4 and its receptors ALK2, ALK3 and ALK6 in human PVR membranes. Double-labelled BMP4 (red, the fourth line), ALK2 (red, the first line), ALK3 (red, the second line) or ALK6 (red, the third line) with cytokeratin or α-SMA (green) is shown on human PVR membranes. The blue signal represents the nuclear staining by DAPI. Yellow or orange signals resulted from the overlay of red and green signals, which indicates co-localisation of BMP4 (the fourth line), ALK2 (the first line), ALK3 (the second line) or ALK6 (the third line) with cytokeratin or α-SMA. Figures were acquired using confocal microscopy. Original magnifications: 630x, oil. Scale bar: 10µm Abbreviations: BMP, bone morphogenetic protein; ALK, activin receptor-like kinase; PVR, proliferative vitreoretinopathy.

To investigate whether BMP4 is involved in the pathogenesis of PVR, we first used immunofluorecense confocal microscopy to examine the expression of BMP4 and its receptors ALK2, ALK3 and ALK6 within the ERM from PVR patients. Figure 1 shows dense BMP4, ALK2, ALK3 and ALK6 immunoreactivity within the ERM. Double-staining further revealed that BMP4, ALK2, ALK3 or ALK6 co-localize with the epithelial cell marker cytokeratin and the mesenchymal marker α-SMA (α-smooth muscle actin). As RPE cells are the only epithelial cells present within ERM^4^, it is expected that many BMP4, ALK2, ALK3 or ALK6-positive cells are derived from RPE or transdifferentiated cells.

### BMP4 is down-regulated, whereas its receptors are up-regulated during TGF-β-mediated EMT in RPE cells

**Figure 2:**
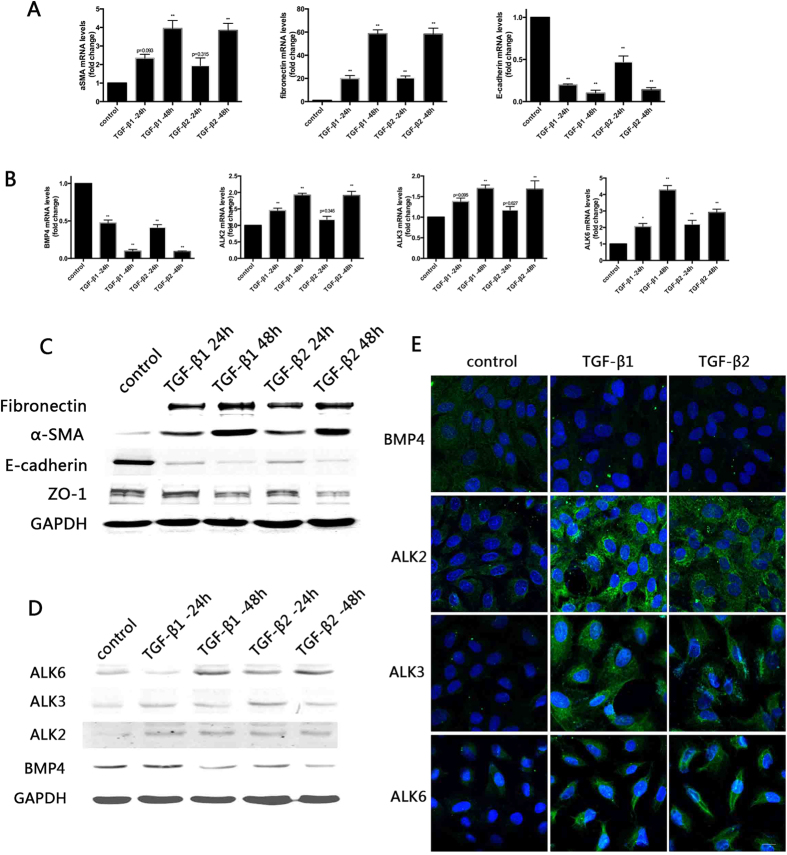
BMP4 is down-regulated, whereas its receptors are up-regulated during TGF-β1 and -b2-induced EMT in RPE cells. Primary RPE cells were treated with transforming growth factors (TGF)-β1 or TGF-β2 (10 ng/ml) for up to 48 hours. (**A**) The mRNA expression levels of the EMT-related markers were detected by RT-PCR. α-SMA, fibronectin and E-cadherin values are shown as the fold-change relative to the control group normalized to GAPDH. The data are presented as mean values ± SEM. N = 3/group. *P < 0.05, **P < 0.01. (**B**) mRNA expression of BMP4, ALK2, ALK3 and ALK6 are shown as the fold-change relative to control normalized to GAPDH. The data are presented as the mean values ± SEM. n = 3/group. *P < 0.05, **P < 0.01. (**C**) EMT marker protein expression levels the EMT markers were detected by Western blot. (**D**) Western blot analysis of ALK2, ALK3 and ALK6 with proteins extracted from primary RPE cells treated with 10 ng/ml TGF-β1 at the indicated time points. GAPDH was used for normalization. (The expanded images of the Western blot with its molecular size and quantification of relative protein expression are shown in the Supplementary Fig. 1A–C. (**E**) Imunofluorescence analysis of BMP4, ALK2, ALK3 and ALK6 (green) in primary RPE cells treated with 10 ng/ml of TGF-β1 at 48 hours. Nuclear were stained with DAPI (blue). Slides were examined by confocal microscopy. Original magnifications: 630x , oil. Scale bar: 10 μm. Abbreviations: BMP, bone morphogenetic protein; ALK, activin receptor-like kinase; EMT, epithelial-mesenchymal transition; TGF: transforming growth factor; α-SMA: α-smooth muscle actin.

The expression pattern of BMP4 and its cognate receptors in PVR membranes suggest that BMP4 might play an important role in the pathogenesis of PVR. As RPE cells, articularly those undergoing EMT play a crucial role in the pathogenesis of PVR, we next investigated whether BMP4 contributes to the EMT process in RPE cells. we used TGF-β1 and TGF-β2 to build an *ex vivo* EMT model. We found that 10 ng/ml TGF-β1 or 10 ng/ml TGF-β2 treatment was sufficient to induced up-regulation of the mesenchymal markers α-SMA and fibronectin and down-regulation of the epithelial markers E-cadherin and ZO-1 at the mRNA (Fig. 2A) and protein (Fig. 2C) levels. Furthermore, the expression of BMP4 during this process decreased after 24 hours of treatment with 10 ng/ml TGF-β1 or TGF-β2. Treatment for 48 hours induced significantly reduced BMP4 expression. However, the expression of the receptors ALK2, ALK3 and ALK6 also increased (Fig. 2B,D). Confocal microscopy (Fig. 2E) revealed that BMP4 staining was down-regulated, whereas that of the cognate receptors ALK2, ALK3 and ALK6 was increased. Furthermore, we found that the receptors were recruited to the cytoplasm and cytomembrane.

### Exogenous BMP4 inhibits TGF-β-induced EMT in RPE cells

**Figure 3:**
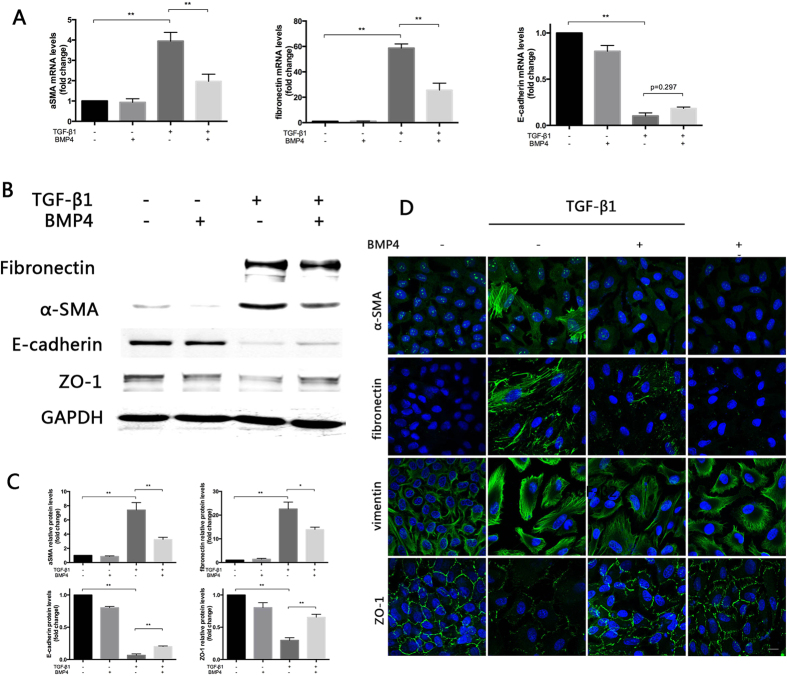
BMP4 treatment attenuates TGF-β1-induced alterations in EMT markers. Primary RPE cells were treated with 50 ng/ml BMP4 and 10 ng/ml TGF-β1for 48 hours. Total RNA and protein were extracted. (**A**) The expression of mRNA levels of α–SMA, fibronectin and E-cadherin were detected with RT-qPCR. The data were presented as the mean values ± SEM. n = 3/group. *P < 0.05, **P < 0.01. (**B**) Primary RPE protein lysates were extracted and subjected to western blot analysis for α-SMA, fibronectin, ZO-1 and E-cadherin was performed. GAPDH was used as the protein loading control. (The expanded images of the Western blot with its molecular size are shown in the Supplementary Fig. 1) (**C**) Quantification of relative protein expression (normalized to GAPDH) in Western blots through determining their grayscale value. The data are presented as the mean values ± SEM. n = 3/group. *P < 0.05, **P < 0.01. (**D**) Immunofluorescence staining of α-SMA, fibronectin E-cadherin and ZO-1 expression in primary RPE cells. (green: the staining of corresponding protein, blue: nuclei staining of DAPI) Original magnifications: 630x, oil. Scale bar: 10 μm.

As a result of the findings detailed above, we were interested in whether exogenous BMP4 could reverse EMT in RPE cells. Because of the similar effects of TGF-β1 and TGF-β2, TGF-β1 was predominantly used in the following experiments. The RT-qPCR results (Fig. 3A) revealed that the TGF-β1-induced expression of mesenchymal markers fibronectin and α-SMA were significantly suppressed significantly by treatment with 50 ng/ml BMP4 respectively (P < 0.05), whereas the expression of the epithelial marker E-cadherin was increased, although not significantly (P > 0.05). Western blot analysis revealed that 50 ng/ml BMP4 attenuated both the TGF-β1 and TGF-β2-induced up-regulation of fibronectin and α-SMA and down-regulation of E-cadherin and ZO-1 in primary RPE cells at the protein level (Fig. 3B,C, Supplementary Fig. 1A). Immunofluorescence confocal microscopy also revealed that intercellular tight junctions were abrogated by TGF-β1 and TGF-β2 and that this process could be rescued by BMP4 (Fig. 3D, Supplementary Fig. 2C).

### BMP4 attenuates RPE collagen gel contraction and TGF-β-induced migration

**Figure 4:**
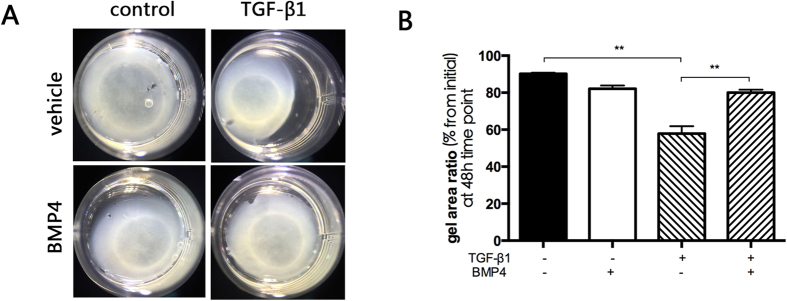
BMP4 suppresses TGF-β1-induced collagen gel contraction mediated by primary RPE cells. Pre-treated primary RPE cells were cultured in the presence or absence of BMP4 and TGF-β1-induced for 1 hour and harvested cells were seeded in a 3D collagen gel system. (**A**) Representative image of collagen gel contraction for 48 hours. (Images of TGF-β1 and -β2-induced collagen gel contraction for 24 or 48 hours are displayed in Supplementary Fig. 2) (**B**) The extent of gel contraction was quantified. The data are presented as the mean values ± SEM that were repeated a total of three times with similar results. **P < 0.01.

**Figure 5:**
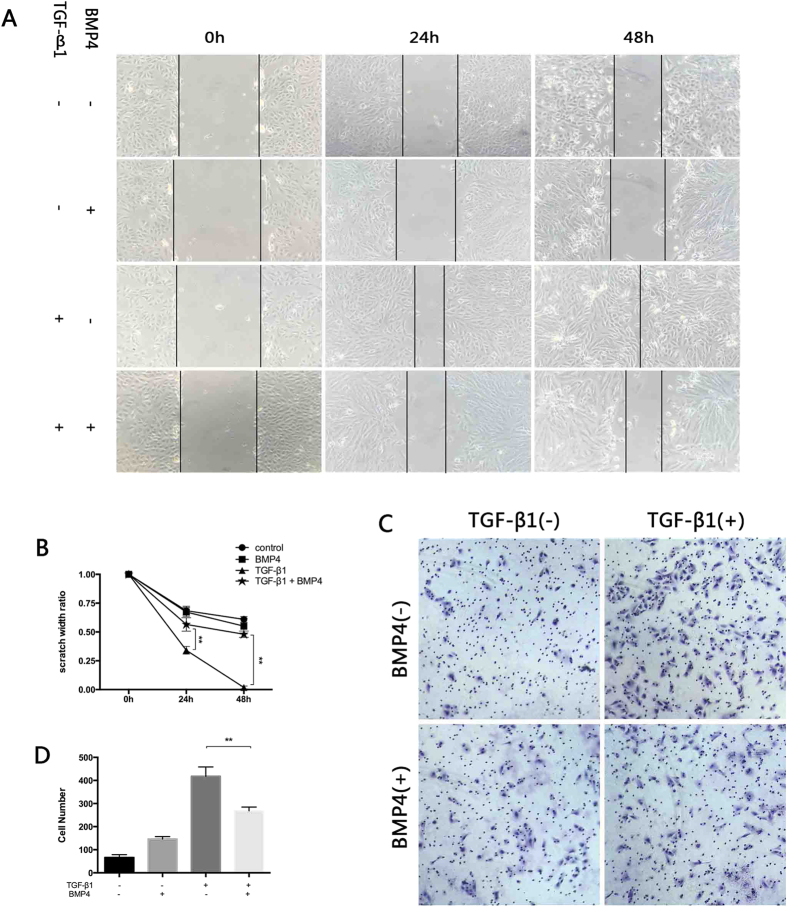
BMP4 treatment attenuates TGF-β1-induced migration of ARPE-19. Pre-treated ARPE-19 was cultured with or without 100 ng/ml BMP4 for 24 hours, after which a scratch was made followed by treatment with10 ng/ml TGF-β1 plus BMP4 for an additional 48 hours. (**A**) The images were taken at 0, 24, and 48 hours after the scratch was applied. Original magnifications: 100x. (**B**) The width of the scratch was measured. The widths at 0 h were used as controls. TGF-β1-induced migration was attenuated significantly by BMP4 in ARPE-19. (**P < 0.01, Dunnett’s test) (**C**) Harvested cells were migrated in the transwell system for an additional 18 hours. Images were acquired under phase-contrast microscopy. Original magnifications: 100x. (**D**) The number of migrated cells. The data shown represent the average of three independent experiments and are presented as the mean values ± SEM. Images of five different fields were taken from each chamber. (**P < 0.01, one-way ANOVA).

Considering that BMP4 attenuated the EMT process of RPE cells at the molecular level, we next examined functional changes by assessing collagen gel contraction and migration. For the contraction assay, primary RPE cells pre-treated with or without TGF-β1 and BMP4 were cultured within a three-dimensional collagen gel. Figure 4 shows that treatment of the RPE cells with 10 ng/ml TGF-β1 resulted in pronounced collagen contraction. However, this effect was suppressed by the 50 ng/ml BMP4 treatment. This contraction effect was time-dependent (Supplementary Fig. 2D). Scratch assays (Fig. 5A,B) demonstrated that 10 ng/ml TGF-β1 enhances the migration and proliferation of RPE cells within the wound area, whereas this capacity was significantly reduced by the 100 ng/ml BMP4 treatment (P < 0.01) in a time-dependent manner. In addition, a modified Boyden chamber assay (Fig. 5C,D) also revealed that BMP4 treatment reduces TGF-β1-induced RPE migration.

### Endogenous BMP4 knockdown promotes EMT in RPE cells

**Figure 6:**
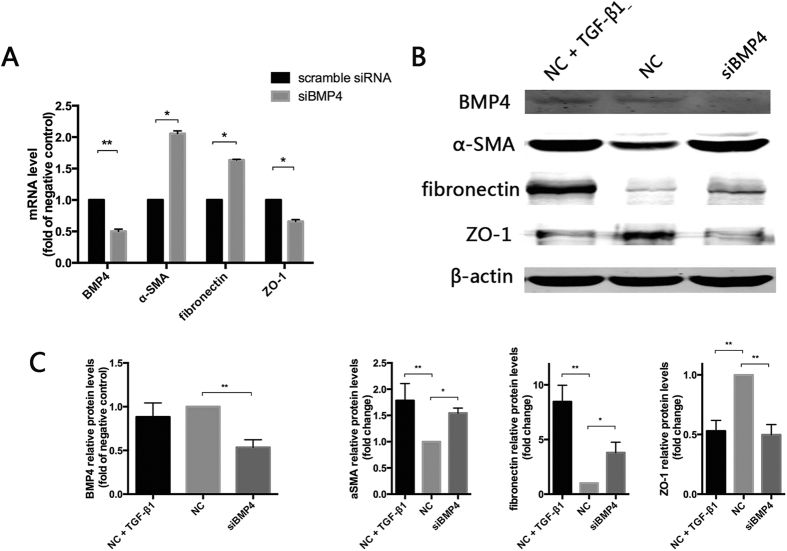
Knockdown of BMP4 increases mesenchymal markers fibronectin and α-SMA but decreases the epithelial marker ZO-1. (**A**) RT-qPCR. The data are presented as the mean values ± SEM. n = 3/group. *P < 0.05, **P < 0.01 (**B**) Representative Western Blot images. β-actin was used as the protein loading control. (**C**) Relative quantification of the Western blots. The data are presented as the mean values ± SEM. n = 3/group. *P < 0.05, **P < 0.01.

In view of the inhibitory effects of exogenous BMP4 on the EMT of RPE cells, we investigated whether knockdown of endogenous BMP4 using specific siRNAs can directly trigger EMT. Compared to scrambled control siRNA-transfected RPE cells, BMP4 siRNA-transfected RPE cells exhibited increased expression of the mesenchymal markers fibronectin and α-SMA at the mRNA (Fig. 6A) and protein (Fig. 6B,C) levels. Moreover, the expression of the epithelial marker ZO-1 was significantly decreased (P < 0.05).

### BMP4 inhibited TGF-β1-induced Smad2/3 phosphorylation via upregulation of Smad1/5/9 phosphorylation

**Figure 7:**
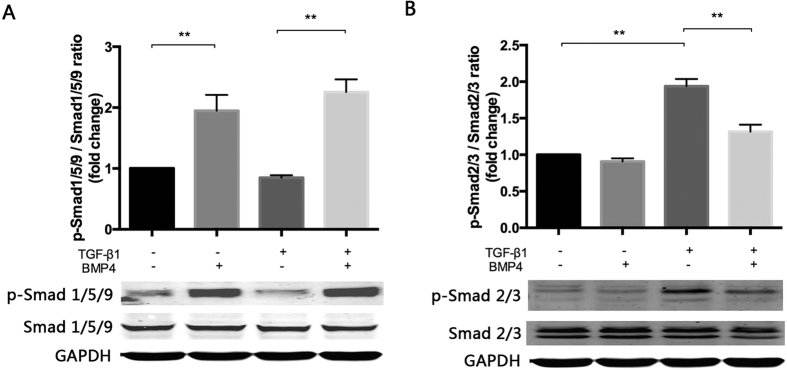
BMP4 treatment attenuates TGF-β1-induced Smad2/3 phosphorylation by increasing activating Smad1/5/9. GAPDH was used as the protein loading control. (**A**) Phosphorylated Smad 1/5/9 and (**B**) Phosphorylated Smad 2/3 was measured by Western blots and semi-quantified with relative total Smad protein. The data are presented as the mean values ± SEM. n = 3/group. *P < 0.05, **P < 0.01.

We further evaluated the effects of BMP4 on TGF-β signalling pathways. Western blot analysis (Fig. 7) revealed that TGF-β1-induced Smad2/3 phosphorylation was attenuated by pre-treatment of 100 ng/ml BMP4 pretreatment. in contrast, the up-regulation of Smad1/5/9 phosphorylation was detected during treatment with BMP4 plus TGF-β1 treatment compared to treatment with TGF-β1 only.

## Discussion

This study demonstrates that BMP4 inhibits EMT by regulating the phosperylation of Smad2/3 and Smad1/5/9. Based on our data, we purpose that BMP4 represents a potential therapeutic agent for PVR.

We first assessed whether BMP4 is involved in the formation of PVR membranes. Surgically excised epiretinal membranes are first-hand specimens; thus, studies using these membranes can more directly represent the pathogenesis of PVR directly and also more closely resemble the disease *in vivo*. Casaroli-Marano *et al*. demonstrated that both epithelial-shaped RPE cells and transdifferentiated RPE cells on the PVR membranes can co-express cytokeratin-vimentin-GFAP^4^. Thus, we used cytokeratin to distinguish RPE cell populations in our research. Double-labelling immunofluorescence microscopy revealed that BMP4-positive cells were derived from RPE cells. α-SMA is a myofibroblast cell marker; thus, co-localization of α-SMA and BMP4 indicated that BMP4-positive cells are derived from transdifferetiated cells. These findings suggest that BMP4 might play a role in the pathogenesis of PVR.

We further examined the expression of BMP4 and its receptors during TGF-β-induced EMT in RPE cells. Three isoforms of TGF-β have been shown to elicit different effects on specific cell types. TGF-β1 and TGF-β2 enhance EMT; whereas TGF-β3 antagonize the effects of TGF-β1 and TGF-β2^27^,^28^. The effects of TGF-β1 and TGF-β2 in PVR are controversial. Some studies have claimed that TGF-β2 plays a crucial role in PVR because the concentration of TGF-β2 is significantly higher compared to that of TGF-β1 within the vitreous^29^. In contrast, other studies have refuted that TGF-β1 is signifcantly up-regulated only during PVR development (and TGF-β2 to a lesser extent). Our research group successfully established a model of RPE cell EMT using TGF-β1^8^. Here, both two isoforms were used to evaluate their roles in EMT in RPE cells. The results confirmed that TGF-β1 and TGF-β2 elicited similar EMT-initiating effects. Our results also revealed the down-regulation of BMP4 and the up-regulation of its receptors during TGF-β1and TGF-β2-induced EMT in primary RPE cells, which implies a potential anti-fibrotic effect of BMP4. Considering the similar effects of TGF-β1 and TGF-β2 on EMT in RPE cells and the predominant use of TGF-β1 in our laboratory, only TGF-β1 was used in the following study. Fibrosis and anti-fibrosis studies of other organs have also focused on the role of BMPs. Some studies have shown that BMP4 enhances cardiac hypertrophy and fibrosis^30^; others have suggested that BMP4 is neuroprotective in Muller glia^31^, stimulated adult RPE apoptosis and inhibited the serum-induced proliferation of RPE cells^32^. Although the role of BMP4 in EMT and fibrosis has been discordant, BMP4 ppears to elicit a protective effect in the eye. The role of BMP4 in the TGF-β1-mediated EMT in RPE cells has rarely been previously reported. Only *Hel Lee et al.* stated that gremlin, the antagonist of BMP4, induced EMT in ARPE-19^33^. To confirm our hypothesis, we added exogenously recombinant BMP4 to our RPE EMT model *in vitro* to determine its functional role. We found that BMP4 significantly attenuates TGF-β1 and TGF-β2-induced increased expression of mesenchymal markers and decreased epithelial markers. In addition, we found that collagen gel contraction and cell migration of RPE cells was reduced in this context. On the other hand, the knockdown of endogenous BMP4 using specific small interfering RNAs (siRNAs) increased the expression mesenchymal markers α-SMA and fibronectin and decreased the epithelial marker ZO-1. The addition of exogenous antagonist gremlin was reported to similarly enhance EMT in ARPE-19^33^. These data provide the basis for the inhibitory effect of BMP4 in EMT.

The contraction of cellular membrane promotes retinal detachment that progressively leads to loss of vision. Thus, contraction can be regarded as a key pathogenic step in PVR. Cells cultivated in the three-dimensional collagen gel system recapitulated similar morphological changes as observed *in vivo*^34^,^35^,^36^. Collagen gel contraction has been used as a model of wound healing process mediated by various cell types, particularly in transdifferentiated RPE cells^37^,^38^,^39^. Because collagen gel contraction efficiently mimics cell contraction, it correlates with PVR *in vitro*. We found that TGF-β-induced RPE contraction was diminished by BMP4. Fibronectin has been reported to contribute to the formation of collagen fibrils and to modulate contraction of fibrotic membranes^40^. Our data also showed that BMP4 treatment down-regulates TGF-β-induced fibronectin expression. These findings prove the theory that BMP4 attenuates the contraction of fibrotic cellular membranes during the pathogenesis of PVR.

Growing evidence implicates a central role for TGF-β1 in EMT of RPE cells. As members of transforming growth factor beta superfamily, TGF-β and BMP4 bind to their receptors to activate key downstream pathways, one of which is the canonical Smad-dependent pathway. The balance of various cytokines and the homeostasis of pathway networks are abrogated by activation of Smad2/3 during EMT^41^,^42^. In our study, this activity was blocked by BMP4 via the activation of Smad1/5/9. These observations support the notion that BMP4 represents a vital factor that inhibits the development of fibrosis.

In conclusion, our results indicate that BMP4 can inhibit TGF-β-induced EMT and gel contraction in RPE cells. Further studies will confirm the protective role of BMP4 in PVR models *in vivo*.

## Methods

### Epiretinal membrane specimens

Epiretinal membranes were acquired from the standard vitreoretinal surgeries of 3 patients diagnosed with retinal detachment complicated with PVR. All patients were fully informed of the purpose of the intraocular surgery and our research study and provided signed consent. Specimens were maintained in iced phosphate buffer saline (PBS, pH 7.4) and fixed in 4% paraformaldehyde for at least 12 hours at 4 °C.

### Cells and cell culture

Human primary RPE cells were isolated from human donors and cultures established according to protocols previously published protocols^43^. The protocol followed the tenets of the Declaration of Helsinki for research involving human subjects. The human RPE cell line ARPE-19 and primary RPE cells were routinely cultured in a 1:1 mixture of Dulbecco’s modified Eagle’s medium and Ham’s F12 medium (DMEM/F-12, Gibco, Carlsbad, CA) supplemented with 10% foetal bovine serum (FBS; Gibco, Carlsbad, CA) and 1% Penicillin-Streptomycin (Carlsbad, CA) at 37 °C in a 5% CO_2_ incubator. The medium was changed every two days. For some experiments, cells were plated equally and cultivated in serum-free medium for 12 hours before stimulation with 10 ng/ml of TGF-β1 or TGF-β2 with or without BMP4 at the indicated concentration.

### SiRNA transfection

Small interfering RNAs (siRNAs) for human BMP4 and a nonspecific control siRNA were purchased from GenePharma (Suzhou, China). ARPE-19 was transfected with 20 nM siRNA using Lipofectamine 3000 (Invitrogen, Carlsbad, CA) according to the manufacturer’s protocol. Transfection efficiency was detected directly using fluorescence microscope (Leica, Wetzlar, Germany) and analyzed by RT-qPCR and Western blot.

### Reagents and antibodies

Human recombinant TGF-β1 and TGF-β2 were purchased from Gibco (Carlsbad, CA) and human recombinant BMP4 was purchased from R&D systems (Inc., Minneapolis, MN, USA). The following antibodies were used for Western blotting and immunofluorescence: BMP4 (Thermo Scientific, Carlsbad, CA), α-Smooth muscle and fibronectin (Sigma-Aldrich, MO, USA), E-cadherin (BD biosciences, San Jose, CA), BMPR1A, BMPR1B, activin receptor type IA, Vimentin, smad1/5/9 and β-actin (Abcam Ltd., Cambridge, USA), ZO-1 (Invitrogen, Carlsbad, CA), phospho-Smad1/5/9, phospho-Smad2/3 and Smad2/3 (Cell Signaling Technology, Danvers, MA, USA).

### RNA extraction and RT-qPCR

Total cellular RNA was extracted by using TRIzol reagent (Invitrogen, Carlsbad, CA) according to manufacturer’s protocol. RNA concentrations were determined using a NanoDrop 2000 spectrophotometer (Thermo Scientific Inc., Carlsbad, CA, USA). Double-stranded cDNA was synthesized from 500 ng total RNA using the PrimerScript RT reagent Kit (TaKaRa Bio Inc., Osaka, Japan) following manufacturer’s instructions. Gene expression was examined by RT-qPCR with the synthesised cDNA and SYBR premix Ex Taq II (TaKaRa Bio Inc., Osaka, Japan). Real-time qPCR was performed using a Bio-Rad System (Hercules, CA). The sequences for RT-qPCR primers are listed in Supplementary Table 1. The thermal cycling conditions included an initial denaturation step at 95 °C for 30 s and 40 cycles of 95 °C for 5 s and 60 °C for 30 s. Relative gene expression was normalized to the level of β-actin mRNA and determined from the data quantified using the comparative threshold cycle (Ct) method^44^.

### Western Blot analysis

Differentially treated cells were lysed using modified RIPA buffer (Beyotime Biotechnology, China) containing a protease inhibitor cocktail and phosphatase inhibitor cocktail (Roche, Mannheim, Germany) on ice for 30 minutes. The lysates were centrifuged at 10,000× *g* for 10 minutes at 4 °C, and the supernatants were collected for further assessment. The protein concentrations were quantified using a BCA Protein Assay Kit (Thermo Scientifics, Carlsbad, CA). Thirty micrograms of total protein were used in the following procedures; loading buffer (100 mM Tris [pH 6.8], 4% SDS, 20% glycerol, 0.02% bromophenol blue, and 50 μl/ml β-mercaptoethanol) were added to each sample, which were then boiled at 100 °C for 10 minutes. The protein samples were concentrated and separated by SDS-PAGE gels according to standard protocols and then transferred onto the hybridisation nitrocellulose membranes (Millipore, Ireland). The membranes were incubated in the blocking buffer (PBST with 5% w/v non-fat dry milk or BSA) for 1 hour at room temperature and in primary antibody dilution buffer with gentle agitation overnight at 4 °C. This procedure was followed by incubation with IRDye 800CW goat anti-mouse IgG(H+L) or IRDye 680LT donkey anti-rabbit IgG(H+L) antibodies (Li Cor Biosciences, NE, USA) for an additional hour at room temperature. The blots were scanned using an Odyssey infrared imaging system (Li-Cor Biosciences, NE, USA).

### Immunofluorescence

Chamber slides of cell cultures pre-treated with the indicated gents were fixed with cold methanol for 1 minute, permeablized with permablized buffer (0.5% Triton X-100 in PBS) for 10 minutes and blocked with blocking buffer (0.2% Triton X-100, 1% BSA in PBS), whereas for epiretinal membrane specimens, divided membranes were fixed in 4% paraformaldehyde and blocked with blocking buffer (0.3% Triton X-100, 1% BSA in PBS). Next, all samples were immunostained with the indicated primary antibodies at 4 °C overnight and the indicated secondary antibodies for 1 hour at room temperature. The slides were mounted with DAPI floromount-G mounting medium (SouthernBiotech Associates, Birmingham, USA) and analyzed using a confocal microscope (Carl Zeiss, LSM710, Jena, Germany).

### Collagen Gel Contraction

Collagen gel contraction assays were performed as previously described^35^,^39^. Briefly, 24-well culture plates were coated with 1 ml 1% BSA for 1 hour at 37 °C. Primary RPE cells, which were pre-treated with TGF-β and BMP4, were harvested and suspended in serum-free DMEM/F12. Collagen I (final concentration, 2 mg/ml; Gibco, Carlsbad, CA), 10× DMEM/F12, cell suspension (final cell density, 2.5 × 10^5^/well), sterile distilled water (ddH_2_O), and sterile 1N NaOH (0.025-fold of volume of collagen) were prepared and mixed on ice. The mixture (total volume, 0.5 ml) was added to BSA-coated well and incubated for 1 hour at 37 °C under 5% CO_2_ to promote polymerization of the gels. The gels were freed from the sides with pipette tips and serum-free DMEM/F12 (0.5 ml) containing the indicated agents was then added on top of the gel. Photographs were taken after 24 or 48 hours to allow for quantitation of the ratio gel contraction area using NIH ImageJ software (version 1.46r; Bethesda, MD, USA).

### Scratch assay and transwell assay

Cells were treated with different agents after scratching in the middle of the well with pipette tips. Light microscope images (Leica, Wetzlar, Germany) were taken every 24 hours to measure the width of scratch. The gap sizes were measured and divided by the original scratch size, and this value was expressed as scratch area ratio. Next, the cells were harvested at the 48-hour time point. Modified Boyden chamber assays were performed as previously reported^45^. A total of 1 × 10^5^ cells in 100 μl of DMEM/F12 containing 0.5% FBS were seeded into the upper compartment of 8-mm pore transwell system (Falcon, corning, Durham, USA), whereas 600 μl of DMEM/F12 containing 10% FBS was added to lower compartment. After an 18-hour-period of migration, non-migratory cells were removed from the upper membrane, while the migratory cells attached to the bottom surface of the membrane were fixed with methanol for 20 minutes and stained with haematoxylin for an additional 20 minutes at room temperature. Images were taken using a phase contrast microscope, and cell counting was performed using NIH ImageJ software.

### Statistical Analysis

All experiments were performed at least three times. All data were collected from at least three similar experiments and presented as the mean values ± SEM. A value of P ≤ 0.05 was considered to be statistically significant. Statistical Analyses were performed by one-way ANOVA, with a Bonferroni correction for multiple comparisons where was applied using the statistical software programme SPSS 20.0 (Chicago, IL). Dunnett’s test was applied for comparisons with the control groups. Graphs in this study were made using GraphPad Prism 6 Software (La Jolla; California; USA).

### Study approval

All procedures used in this study were approved by the Medical Ethics Committee of Shanghai Tenth People’s Hospital. Principles of human subject research and cell research were conducted in accordance with the Declaration of Helsinki. Informed consent was obtained from all patients from whom specimens were procured.

## Additional Information

**How to cite this article**: Yao, H. *et al*. Inhibitory Effect of Bone Morphogenetic Protein 4 in Retinal Pigment Epithelial-Mesenchymal Transition. *Sci. Rep.* **6**, 32182; doi: 10.1038/srep32182 (2016).

## Supplementary Material

Supplementary Information
