# Supplementary material for: Inhibitory Effect of Bone Morphogenetic Protein 4 in Retinal Pigment Epithelial-Mesenchymal Transition
Source: Sci Rep. 2016 Sep 2;6:32182. doi: 10.1038/srep32182 (PMC5009382; doi:10.1038/srep32182)
Supplement: Supplementary Information [file srep32182-s1.pdf]

Inhibitory Effect of Bone Morphogenetic Protein 4 in Retinal Pigment Epithelial-Mesenchymal Transition

Haipei Yao[1,2], Hui Li[1], Shuai Yang[1,2], Min Li[1], Chun Zhao[1], Jingfa Zhang[2], Guotong Xu[1,2]*, and Fang Wang[1,2]*

[1]Department of ophthalmology, Shanghai Tenth People's Hospital, Tongji University, School of Medicine, Shanghai, China

[2]Tongji Eye Institute, Tongji University School of Medicine, Shanghai, China

*Corresponding author

Supplementary Material

Supplementary information contains: Supplementary Table, Supplementary Figures and Legends.

Supplementary Table 1. Primer sequences for real-time qPCR

Targets	Accession	Primers	Sequences
BMP4	NM_130851.2	forward	CCTGGATGAGTATGATAAGGTGGTA
		reverse	TGTGTAGTGTGTGGGTGAGTGGA
ALK2	NM_001111067.2	forward	CATCAAAACCACCCTAACCTCGCTC
		reverse	TCTCTGTGTTCCTCCAGTCCCTACC
ALK3	NM_004329.2	forward	CTATTTGTTCATCATTTCTCGTGTT
		reverse	CTCTGGTGCTAAGGTTACTCC
ALK6	NM_001256794.1	forward	TACAAATGAAGTTGACATACCACCT
		reverse	CCTCCTGATACACATCTCCTAGC
GAPDH		forward	CGCTGAGTACGTCGTGGAGTC
		reverse	GCTGATGATCTTGAGGCTGTTGTC
E-cadherin	NM_004360.3	forward	TCACGCTGTGTCATCCAACGG
		reverse	TAGGTGTTCACATCATCGTCCGC
ZO-1	NM_003257.3	forward	AGCCATTCCCGAAGGAGTTGAG
		reverse	ATCACAGTGTGGTAAGCGCAGC
Vimentin	NM_003380.3	forward	AATCCAAGTTTGCTGACCTCTCTGA
		reverse	ACTGCACCTGTCTCCGGTACTC
α-SMA	NM_001141945.1	forward	CCTTGAGAAGAGTTACGAGTTGC
		reverse	ATGATGCTGTTGTAGGTGGTTT
Fibronectin	NM_212482.1	forward	AAGACCATACCCGCCGAATG
		reverse	GGCATTTGGATTGAGTCCCG

Abbreviations: ALK: activin receptor-like kinase; ZO-1: zona occludens 1; α-SMA: α-smooth muscle actin

Supplementary Figures and Legends

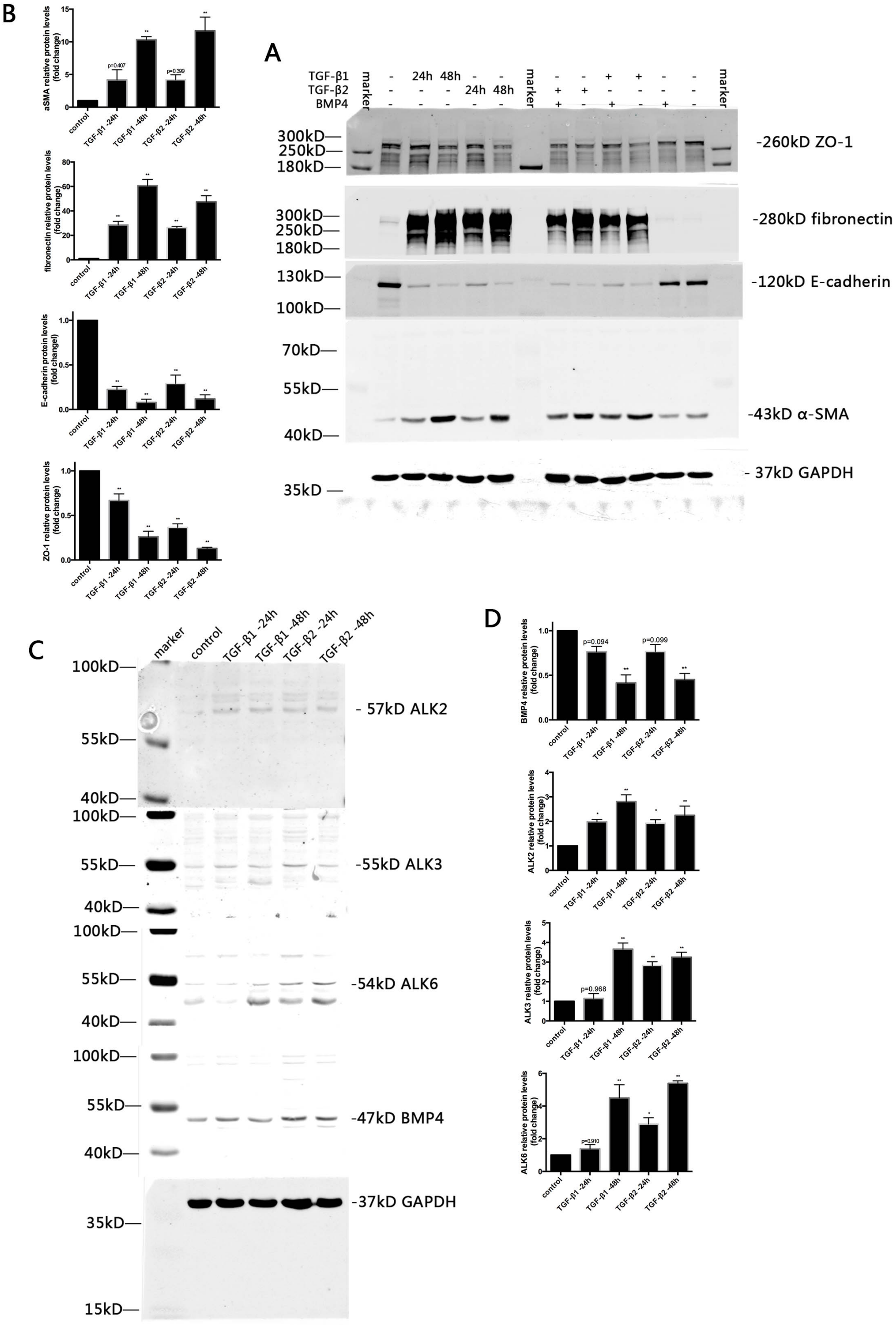

Supplementary Fig. 1 BMP4 is down-regulated, wheraes its receptors are up-regulated during TGF-β1 and -b2-induced EMT in RPE cells. (A) This figure represent the expanded NC membrane images of the western blot analysis for the original Fig. 2C and 3B. (B) Semi-quantification for the western blots in original Fig. 2C are shown as fold-change relative to control normalized to GAPDH. The data are presented as the mean values ± SEM. n=3/group. *P<0.05, **P<0.01. (C D) Expanded images and semi-quantification for original Fig. 2D. They are shown as fold-change relative to control normalized to GAPDH. The data are presented as the mean values ± SEM. n=3/group. *P<0.05, **P<0.01.

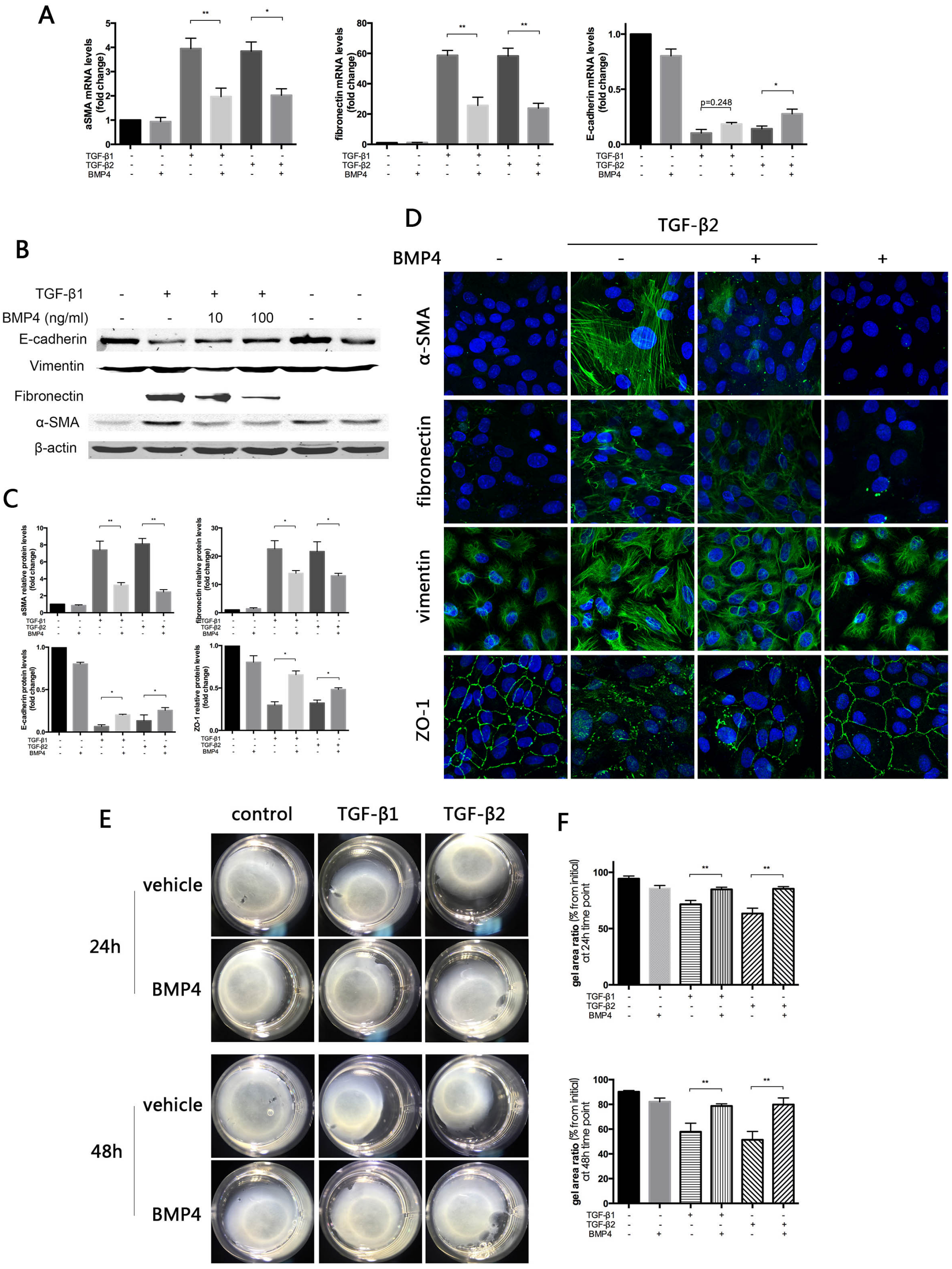

Supplementary Fig. 2 BMP4 treatment attenuates TGF−β1-induced alterations in EMT markers. Primary RPE were treated with 50 ng/ml BMP4 and 10 ng/ml TGF-β1or TGF-β2 for 48 hours. Total RNA and protein were extracted. (A) The expression of mRNA levels of a-SMA, fibronectin and E-cadherin were detected with RT-qPCR. The data are presented as the mean values ± SEM. n=3/group. *P<0.05, **P<0.01. (B) Representative images of Western blots of ARPE-19 treated with

BMP4 and TGF-b1. (C) Semi-quantification for the western blot analysis in original Fig. 3 and supplementary Fig. 1A (adding TGF-β and BMP4 part) are shown as fold-change relative to control normalized to GAPDH. The data are presented as the mean values ± SEM. n=3/group. *P<0.05, **P<0.01. (D) Immunofluorescence microscopy staining of α-SMA, fibronectin E-cadherin and ZO-1 expression in primary RPE cells treated with or without TGF-β2 and BMP4. (green: the staining of corresponding protein, blue: nuclei staining of DAPI) Original magnifications: 630×, oil. Scale bar: 10μm. (E F) Images of TGF-β1 and –β2-induced collagen gel contraction images and the quantification of contractile area. The data are presented as the mean values ± SEM that were repeated a total of three times with similar results. **P<0.01
